# Gut-Lung Axis in COVID-19

**DOI:** 10.1155/2021/6655380

**Published:** 2021-03-12

**Authors:** Imane Allali, Youssef Bakri, Saaïd Amzazi, Hassan Ghazal

**Affiliations:** ^1^Laboratory of Human Pathologies Biology, Department of Biology, Faculty of Sciences, and Genomic Center of Human Pathologies, Faculty of Medicine and Pharmacy, Mohammed V University in Rabat, Rabat, Morocco; ^2^National Center for Scientific and Technical Research, Rabat, Morocco; ^3^School of Medicine, Mohammed VI University of Health Sciences, Casablanca, Morocco

## Abstract

COVID-19 is a pandemic infection of the respiratory system caused by severe acute respiratory syndrome coronavirus 2 (SARS-CoV-2). The viral ribonucleic acid (RNA) was found in many parts of the COVID-19 patients including the stool, suggesting a potential interaction with the host's gut microbiome. The gut microbiome also plays major roles in immunity and inflammation. It also impacts pulmonary functions through the gut-lung axis. There have been recent reports of the importance of the host microbiome in infection and pathogenicity. The understanding of the gut and lung microbiomes would open the gate to new therapeutic approaches.

## 1. Introduction

The world is facing a new pandemic infection of the respiratory system caused by severe acute respiratory syndrome coronavirus 2 (SARS-CoV-2) named “COVID-19” [1]. It started first in December 2019 in Wuhan, China, and it has spread globally affecting more than 213 countries with more than 11 million confirmed cases around the world by March 1, 2021. In the twenty-first century, two previous coronavirus outbreaks have occurred which are SARS-CoV and Middle East respiratory syndrome (MERS)-CoV with almost the same clinical symptoms of SARS-CoV-2 [2–5]. SARS-CoV and SARS-CoV-2 viruses attach to the angiotensin-converting enzyme 2 (ACE2) receptor, which is considered as an entry point to the host cell. This ACE2 receptor is expressed in different organs such as the lungs, the gastrointestinal tract, the heart, and the kidney that make them all a target of coronaviruses [6]. Primarily, SARS-CoV-2 infects the respiratory tract and can cause severe respiratory infections. However, coronavirus viral RNA was detected in the stool of COVID-19 patients and can also cause gastrointestinal infections [7–11]. These findings suggest the importance of considering the microbiome and the gut-lung axis to better understand the characteristics and the mechanisms of COVID-19 infection and pathogenesis [12–15].

Over the last decade, several studies showed the pivotal role of the microbiome and particularly the gut microbiome in health and diseases [16, 17]. Until recently, many microorganisms (bacteria, viruses, and fungi) have also been identified in the respiratory tract rendering the concept of the presence of these microbes in the lungs as pathologically completely wrong [18]. The lung microbiome is still less studied compared to the gut, the skin, and the urogenital tract. This is due to many difficulties, such as the access by invasive sampling (bronchoalveolar lavage) and oropharyngeal contamination. Given the increasing number of cases with respiratory diseases or infections [19–21], more researchers are investigating the role of the lung microbiome and the crosstalk between the gut and the lung in the pathogenesis of these diseases.

In this study, we highlighted the major role of microbiome considering the interactions between the microbial communities in the gut and the lung and also the importance of the gut-lung axis in the immune response in the context of COVID-19. Finally, we pointed out the potential new therapeutic approaches offered by the use of probiotics.

## 2. Lung Microbiome

For many years, the lung has been considered a sterile and aseptic organ. Recently, thanks to the development of Next-Generation Sequencing (NGS) techniques, we know that this is not the case and that microorganisms inhabit the lung and form its local microbiome like the gut microbiome [18, 22]. Although the number of microorganisms found in the lung is not as high as in the intestine or oral cavity, the lung microbiome is now considered as a specific ecosystem where the diversity of different microorganisms composing it is unique for each individual [23, 24]. The lung microbiome is a more dynamic and transient ecosystem; its microorganisms come mainly from the oral sphere and also from inhaled air and the digestive tract (by microaspiration). The most frequent phyla found in the lung microbiota composition are Proteobacteria, Firmicutes, and Bacteroidetes, while at the genus level, *Pseudomonas*, *Streptococcus*, *Prevotella*, *Fusobacteria*, *Porphyromonas*, and *Veillonella* are the most predominant according to different studies [25–27].

With the emergence of lung microbiome research, many studies focused on the comparison between the bacterial communities of the lungs in healthy subjects and those of patients with chronic pulmonary diseases such as asthma, chronic obstructive pulmonary disease (COPD), cystic fibrosis, fibrosis idiopathic lung disease, and lung cancer [19, 20, 27–29]. The associations between the lung microbiome composition and asthma have been reported in different studies [29, 30]. Two main studies on large cohorts of children have demonstrated the impact of the child environment and the disturbances of the growth-accumulating lung microbiome composition on the risk of developing asthma [31, 32]. Moreover, viral infections, in particular with *rhinovirus,* could play a role in the genesis of asthma [33, 34]. In addition, differences in bacterial communities were observed between asthmatic and healthy children. Proteobacteria and *Staphylococcus* were overrepresented in children with asthma, while Bacteroidetes, Firmicutes, and Actinobacteria are more in healthy children [29, 30]. Recent studies suggest that the more diverse the lung microbiota, the lower the risk of developing asthma [34]. Conversely, colonization by specific bacteria would increase the occurrence of asthma [33].

In recent years, the associations between lung microbiome and COPD have been the subject of several studies. A study comparing the microbiome of 9 COPD patients and 9 healthy subjects showed differences in bacterial communities between the two groups [35]. Another study investigated the effects of *rhinovirus* infection on the lung microbiome [36], in which subjects with COPD and healthy control individuals were infected with *rhinovirus* and their microbiomes were analyzed at different time points. The results of this study showed a decrease in bacterial diversity in healthy subjects, while in COPD patients, an increase of *Haemophilus influenzae* which already exists in their lung microbiome has been observed. These findings explained the impact of viral infections on the respiratory microbiome and its potential implication in secondary bacterial infections [36].

In addition, the lung microbiome plays a role in regulating the immune response like the gut microbiome [37, 38]. Indeed, a loss of the diversity of the pulmonary microbiome would cause an imbalance in immunological homeostasis which could have an impact on the genesis of chronic inflammatory respiratory diseases [19, 24]. Nowadays, the associations between lung microbiome and several respiratory diseases have been shown, but the role of the respiratory microbiota is still poorly understood.

## 3. Gut-Lung Axis

Over the past few years, many studies revealed the vital role of the gut microbiome composition in our health [16, 17]. The important physiological role of the gut microbiota is recognized to the point to be considered as the “neglected organ” that is essential to preserve and understand its impact on health and diseases [39]. An imbalance in our gut microbiome is the cause of many diseases, including metabolic diseases [40], neurodegenerative disorders [41], noncommunicable diseases [42], and infectious diseases [43]. However, the impact of the microbiome in the etiology of these diseases remains not yet elucidated because of the complexity of the gut microbiome composition. At birth, the human gastrointestinal tract is colonized by a rapidly developing microbial ecosystem. Although the microbiota is formed early in life, it can change during its existence, with changes related to age, diet, geographic location, antibiotic consumption, and other environmental influences [44–46]. Microbial communities undoubtedly play an important role in human development, physiology, immunity, and nutrition [17, 20, 45, 46].

It is well known that the gut microbiome plays a major role in initiating, adapting, and regulating the immune response [47, 48]. The gut microbiome produces a certain number of metabolites, such as short-chain fatty acids (SCFAs) [49]. SCFAs are products of the fermentation of carbohydrates by anaerobic bacteria present in the colon. They have anti-inflammatory effects such as inducing apoptosis, inhibiting the cell cycle of tumor cells, and preserving the mucosal barriers to endotoxin infiltration [50]. Since most of the immune cells are found in the intestine, the gut microbiome plays an important role in the immunity of the gut as well as in the immune response of other organs. Nowadays, there is growing evidence for the important link between the gut microbiome and other organs such as the brain, the liver, the heart, and the lung [13, 51–54]. Emerging studies have highlighted the evidence for a crosstalk between the gut and the lung microbiomes that refers to the gut-lung axis [13]. Changes in the gut microbiome composition are associated with an increase in susceptibility to respiratory diseases and modifications in the immune responses and homeostasis of the lungs [55].

In fact, many studies have shown that patients with chronic gastrointestinal diseases such as inflammatory bowel disease (IBD) also have a higher prevalence of pulmonary diseases [56–58]. Moreover, a recent study reported the association of fecal microbiota with bronchiolitis in infants where the authors identified four distinct fecal microbiota profiles (*Escherichia*, *Bifidobacterium*, *Enterobacter*/*Veillonella*, and *Bacteroides*) as dominant taxa and also the association of *Bacteroides* with a higher likelihood of bronchiolitis [59]. In addition, several studies have linked the impact of antibiotics use in early life with disturbances on the gut microbiome and an increased risk of asthma [60, 61]. Fujimura et al. showed a lower relative abundance of *Bifidobacterium*, *Akkermansia*, and *Faecalibacterium* in the human neonatal gut microbiome, which are linked to an increased risk of childhood atopy and asthma [47]. A recent study in mice revealed that disturbances in the gut microbiome caused by the influenza A virus enhanced secondary bacterial infections and highlighted the importance of SCFAs on the host's pulmonary defenses against bacterial infections [62]. Moreover, Wang et al. found that respiratory influenza infection caused intestinal injury and changed the gut microbiome composition with an increase in Enterobacteriaceae and a decrease in *Lactobacillus* and *Lactococcus* [63]. The crosstalk between the gut and the lung is established, but the mechanisms through which the gut influences the lung or vice versa are not yet understood and still in their beginnings.

## 4. Importance of the Gut Microbiome in COVID-19

Since the gut microbiome is associated with many respiratory diseases and plays a major role in immunity, better understanding of the new coronavirus infection is needed. COVID-19 is primarily an infection of the respiratory system caused by severe acute respiratory syndrome coronavirus 2 (SARS-CoV-2) [1]. These coronaviruses are viruses from the Coronaviridae family; they can be pathogenic in humans and animals and cause respiratory infections whose manifestations range from the common cold to more serious illnesses. However, coronavirus can also cause gastrointestinal infections which make the gut not really spared from COVID-19 [7, 64].

In fact, many COVID-19 patients also suffer from gastrointestinal disorders such as diarrhea and nausea, which can occur before respiratory conditions [7, 9]. According to a Chinese study, Pan et al. analyzed laboratory, imaging, and historical data of 204 patients and found that almost half of these patients reported digestive symptoms, mainly diarrhea, vomiting, and abdominal pain [7]. In addition, some studies revealed viral RNA was detected in the stool of patients and even the presence of live viruses [8, 10, 65]. Since the coronavirus was found in fecal samples, a potential transmission by the fecal-oral route could be suggested [66]. Moreover, the authors of another study have analyzed the gut microbiome of COVID-19 patients and found that *Bifidobacterium*, *Lactobacillus*, and *Eubacterium* were significantly reduced while pathogenic bacteria such as *Corynebacterium* (Actinobacteria) and *Ruthenibacterium* (Firmicutes) were significantly increased in affected patients [67]. A recent study suggests that the gut microbiome could underlie the predisposition of healthy individuals to COVID-19. They have first analyzed blood proteomics data from COVID-19 patients and found that 20 proteomic biomarkers may be linked to the severity of the disease based on the proteomic risk score (PRS) for predicting the progression of COVID-19 [14]. Using a machine learning model, they have also linked the gut microbiota to COVID-19 severity and found a core of bacteria that are associated with inflammation, such as *Ruminococcus* and *Blautia* that are positively associated while *Bacteroides* and *Clostridiales* are negatively associated. All these studies shed light on the implication of the gut microbiome in the SARS-CoV-2 infection and its importance in a better understanding of the characteristics and the mechanisms of COVID-19. In addition, it has been well known that the diversity of the gut and the lung microbiomes decrease with age leading to a weaker and imbalanced immune system [68]. These two factors could correlate with the increase in the number of cases in older individuals and the severity of COVID-19 in this age category.

Otherwise, considering our knowledge of the lung microbiome and its associations with the gut microbiome in respiratory diseases, including COVID-19, this might open new potential therapeutic approaches, thanks to the use of probiotics [15]. Thus, the administration of specific strains such as *Lactobacillus* spp. in the respiratory and/or digestive tract could play a protective role against some pathologies such as cystic fibrosis or nosocomial pneumonitis [69]. Liu et al. have recently demonstrated that recombinant *Lactobacillus plantarum* contains antiviral effects against coronavirus infection in the epithelial cells of the intestine in an animal model [70]. Many studies reported the benefits of *Lactobacillus rhamnosus* GG in maintaining intestinal and lung barrier homeostasis, reducing apoptosis, increasing regulatory T cells, and reducing proinflammatory cytokines in the gut and the lung [71, 72]. Another study supported the use of probiotics in COVID-19 since little is known about the disease, and it has been well documented that probiotics have antiviral effects [73]. However, the effects of probiotics in reducing the mortality and the severity of COVID-19 are still not proved [74]. More studies on the effects of probiotics and prebiotics on COVID-19 should be conducted.

## 5. Conclusion and Perspectives

In this global circumstance of COVID-19, scientific researchers are dedicated to finding effective ways to understand, prevent, and treat viral infections, which might help to overcome SARS-CoV-2 pathogenicity. Although COVID-19 is primarily a respiratory infection, it also affects the gastrointestinal tract [9, 11]. It has been well documented that most chronic diseases such as obesity, diabetes, hypertension, and heart disease are linked to gut dysbiosis [40, 49, 75] and also to COVID-19 complications [10, 14], making the gut microbiome an essential part to consider in understanding COVID-19. Over the past few years, microbiome research, thanks to NGS technologies, allowed us to better understand the role of gut, mouth, urogenital, and lung microbiomes in health and disease [17, 37, 48]. Nowadays, one of the major challenges is to decipher the implications of these microbes on our immune system. However, viruses are less studied in comparison to the bacterial community, which makes our understanding of the microbial communities less global because of a lack of characterization of one of its components. Moreover, human virome is still underestimated, but it could explain more the dynamic of the microbiome. Especially, some studies have shown the associations between viruses and diseases such as the case of COVID-19. With the major environmental disorders that mankind has generated during many decades, we are now more conscient that these microbes can become extremely pathogenic at any time. Regarding all of these factors, more microbiome studies in analyzing bacterial and viral communities to demonstrate potential associations between microbial communities, virome, and diseases are needed.

In the context of COVID-19, it will be interesting to perform an integrative approach that combines omics data to understand the complexity of such biological systems. A combined microbiome data from different body sites including the nasopharynx, mouth, and gut in COVID-19 patients at different stages of infection (asymptomatic, mild, or severe symptoms) should be studied. All of these data will allow us to define microbiome signatures in COVID-19 patients that could be considered as potential taxonomic biomarkers capable of predicting the occurrence of the disease and its evolution. In addition, some studies suggested using probiotics to balance the gut microbiome composition towards a more diverse ecosystem that could strengthen our immune system and regulate the inflammation that is one of the first symptoms of COVID-19. More studies on the gut-lung axis will allow for a deeper understanding of the interactions between the host, the gut, and the lung microbial communities in respiratory diseases or infections for new therapeutic approaches.

## Data Availability

No data were used to support this study.
